# Improving somatic exome sequencing performance by biological replicates

**DOI:** 10.1186/s12859-024-05742-5

**Published:** 2024-03-22

**Authors:** Yunus Emre Cebeci, Rumeysa Aslihan Erturk, Mehmet Arif Ergun, Mehmet Baysan

**Affiliations:** Department of Computer Engineering, Istanbul Technical University, 34469 Istanbul, Turkey

**Keywords:** Replicate-based consensus, Somatic sequencing, Single nucleotide variant, Next generation sequencing, Whole exome sequencing, SEQC2, Machine learning, NeuSomatic ensemble, Cancer genomics, Precision medicine

## Abstract

**Background:**

Next-generation sequencing (NGS) technologies offer fast and inexpensive identification of DNA sequences. Somatic sequencing is among the primary applications of NGS, where acquired (non-inherited) variants are based on comparing diseased and healthy tissues from the same individual. Somatic mutations in genetic diseases such as cancer are tightly associated with genomic instability. Genomic instability increases heterogenity, complicating sequencing efforts further, a task already challenged by the presence of short reads and repetitions in human DNA. This leads to low concordance among studies and limits reproducibility. This limitation is a significant problem since identified mutations in somatic sequencing are major biomarkers for diagnosis and the primary input of targeted therapies. Benchmarking studies were conducted to assess the error rates and increase reproducibility. Unfortunately, the number of somatic benchmarking sets is very limited due to difficulties in validating true somatic variants. Moreover, most NGS benchmarking studies are based on relatively simpler germline (inherited) sequencing. Recently, a comprehensive somatic sequencing benchmarking set was published by Sequencing Quality Control Phase 2 (SEQC2). We chose this dataset for our experiments because it is a well-validated, cancer-focused dataset that includes many tumor/normal biological replicates. Our study has two primary goals. First goal is to determine how replicate-based consensus approaches can improve the accuracy of somatic variant detection systems. Second goal is to develop highly predictive machine learning (ML) models by employing replicate-based consensus variants as labels during the training phase.

**Results:**

Ensemble approaches that combine alternative algorithms are relatively common; here, as an alternative, we study the performance enhancement potential of biological replicates. We first developed replicate-based consensus approaches that utilize the biological replicates available in this study to improve variant calling performance. Subsequently, we trained ML models using these biological replicates and achieved performance comparable to optimal ML models, those trained using high-confidence variants identified in advance.

**Conclusions:**

Our replicate-based consensus approach can be used to improve variant calling performance and develop efficient ML models. Given the relative ease of obtaining biological replicates, this strategy allows for the development of efficient ML models tailored to specific datasets or scenarios.

**Supplementary Information:**

The online version contains supplementary material available at 10.1186/s12859-024-05742-5.

## Introduction

A DNA sequence consists of an ordered set of nucleotides [1]. It is inherited from parents and establishes the continuity of species [2]. Recent improvements in sequencing technologies have enabled detailed profiling of DNA sequences at reduced costs and within shorter timeframes [1]. An obvious target for these technologies was the familial diseases for which the responsible locus was unknown. Today, we know both the responsible loci and the molecular mechanisms for many such diseases through germline sequencing [3].

Cancer is defined as uncontrolled cell growth and is a major threat to human health, which causes millions of deaths each year [4]. Conventional methods used to cure cancer, like chemotherapy and radiation, not only inflict considerable harm on non-tumor cells but also profoundly affect patient’s quality of life [5]. Understanding the molecular changes that lead to cancer is crucial to develop efficient targeted therapies [6]. Heterogeneity among the patients within a cancer type and among different cancers requires detailed profiling of genomic changes for each patient [7]. For many genetic diseases, including cancer, both inherited-germline and acquired-somatic variants are important [8]. While germline and somatic sequencing share similarities, significant distinctions also exist between the two [9]. In germline sequencing, the sequence of target samples is compared against the (global) reference genome, while somatic sequencing involves the comparison of diseased and non-diseased (healthy) samples from the same individual.

Currently, almost all of the sequencing technologies are based on short reads of DNA with sizes ranging from fifty to a few thousand nucleotides [10]. These reads are not perfect, and error rates increase with read length [10]. These reads are mapped to the global reference genome, allowing an imperfect match considering the differences between the reference genome and the target sample. Here, the number of possibilities is too high to cover considering the size of the genome therefore, heuristics are developed to find near-optimal solutions for reasonable time and resources [11]. In addition to read errors, frequent repeats and transposable elements in the human genome make mapping more difficult. Structural events such as inversions, indels, and copy number changes in cancer samples make somatic sequencing even more problematic. Finally, variants are detected based on the reference and alternative reads that are mapped to the same locus [12]. For germline variants, this is relatively straightforward since variant frequency (portion of alternative reads) is expected to be 0.5 (half of the reads) for heterozygous, and 1 (all of the reads) for homozygous variants. On the other hand, in somatic variants, contamination and heterogeneity decrease the alternative allele frequencies and make reliable detection of complex variants extremely challenging.

These difficulties lead researchers to develop solutions to improve the reliability of variant detection. As the ultimate solution, variants can be validated through manually targeted approaches such as PCR and Sanger sequencing [13]. Due to its cost and time-intensive nature, this process can only be feasibly conducted for a limited number of variants within each sample. Another approach is using ML to detect reliable variants [14]. ML models require training data from samples with known variants. Ideally, the training and test data should closely resemble each other for effective model training. Unfortunately, the number of such training samples is very limited, especially for somatic sequencing [15]. Heterogeneity among cancer datasets makes it very difficult to find appropriate training sets. Scientists have developed simulation frameworks to create in silico variants to construct training datasets for different conditions such as alternative read depth, heterogeneity, and purity levels [16]. Nonetheless, creating realistic variants and proving their equivalence to real variants are also very challenging tasks.

Recently, large-scale benchmarking studies have been developed to overcome this major problem. Broader use and comparative simplicity made germline sequencing the major target of benchmarking and reproducibility studies such as GIAB [17]. Such studies are scarce for somatic sequencing, even though there is a greater demand for them. SEQC2 consortium [18] is an important exception. Their recent publications include datasets for detailed profiling of both non-tumor and tumor cell lines that belong to the same patient [15, 19]. In addition to raw data with many replicates, which are obtained from various centers and platforms, they also include high-confidence regions and high-confidence variants for benchmarking.

**Fig. 1:**
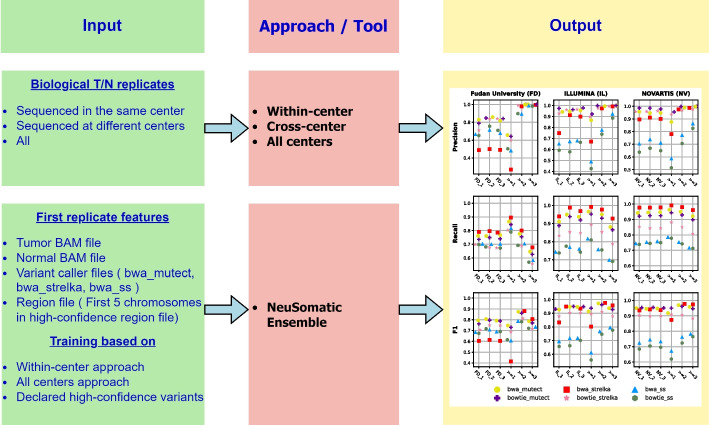
Overview of the study. This study used 12 somatic fresh SEQC2 T/N (Tumor/Normal) biological replicates sequenced at six centers. Three centers have three replicates, and three have one replicate. We developed three replicate-based consensus approaches (within-center, cross-center, and all centers) using the results of three replicate centers. In the within-center approach, we used replicates from the same center; in the cross-center approach, we used replicates from different centers; and in the all centers approach, we used the results of all replicates as input. We accepted the declared high-confidence variants as ground truth and extracted the precision, recall, and F1 scores of these approaches. In the second part of the study, we trained machine learning models based on the results of these approaches (detected somatic variants). We used the information on the first five chromosomes in the training set and the remaining chromosomes in the test set. We also trained machine learning models based on declared high-confidence variants (instead of developed approaches). Finally, we extracted the precision, recall, and F1 score of these trained machine learning models by accepting declared high-confidence somatic variants as ground truth

We aimed to enhance variant calling performance and develop ML models using different replicate-based variant lists in this study (Fig. 1). To address this question, we examine the effect of combining multiple biological replicates on variant calling performance. In pursuit of this goal, we employed the latest datasets from the SEQC2 consortium. We conducted comparisons to assess the potential gains achieved on variant calling performance by utilizing replicates sourced from the same center as well as replicates obtained from different centers. We focused on whole exome sequencing (WES) samples, considering the wider use and more straightforward interpretation for coding regions. We have processed the available exome samples on two mappers (*bwa* [20], *bowtie 2* [21]) and three variant callers (*Mutect2* [22], *Strelka2* [23], and *SomaticSniper* [24]). Then, we developed replicate-based consensus (multiply-detected variants) approaches for the pipelines in the following scenarios: (1) among replicates in the same center (within-center), (2) among replicates from different centers (cross-center), and (3) replicates from all centers (all centers). We compared the detected variants with high-confidence variants in high-confidence regions for performance evaluation. Finally, we trained ML models using the multiply-detected variants as labels in the training set. We performed all ML experiments with the NeuSomatic [25] package in ensemble mode. Subsequently, we evaluated the performance of these ML models against those trained using previously declared high-confidence variants.

## Results

### SEQC2 exome dataset and somatic pipeline used

**Fig. 2:**
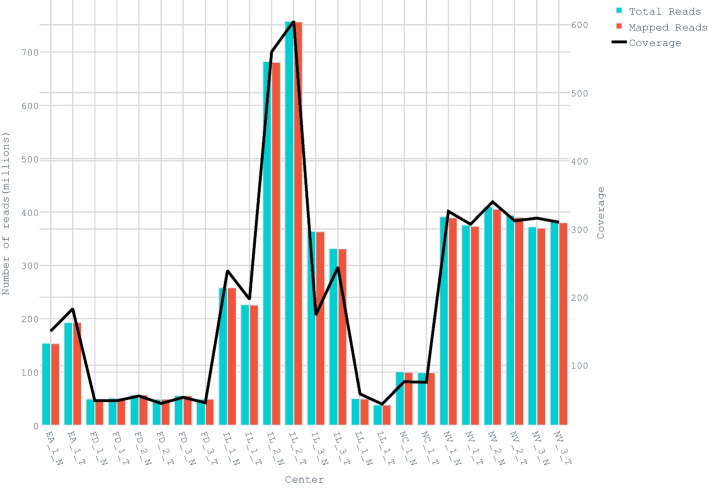
Read and mapping statistics of 12 SEQC2 exomic replicates for 6 centers. X-axis shows the center name, replicate number, and sample type (T: tumor & N: normal) of replicates, separated by underscores. For this calculation, the Qualimap bamqc tool was used in the high-confidence exome region of the marked duplicates BAM files. For the number of reads and mapped reads, we used their global values in Qualimap, while for coverage, we used the mean coverage inside of regions value. Marked duplicates BAM files were generated as an input for Qualimap resulting from the following steps in order in the pipeline: trimming, bwa mapping, sorting, indexing, and marked duplicates

SEQC2 exome dataset includes FASTQ files from tumor and normal samples of the same individual. These samples were sequenced across six different centers and encompassed a total of 12 replicates. Among the centers; European Infrastructure for Translational Medicine (EA), Loma Linda University (LL), and National Cancer Institute (NC) have one replicate, while centers Fudan University (FD), Illumina (IL), and Novartis (NV) have three replicates. While the majority of germline variants are at 50% and 100% variant frequencies in a given sample, somatic variants may be at lower frequencies due to intra-sample heterogeneity. Therefore, coverage is a crucial factor in somatic variant detection. Figure 2 shows the number of reads, coverage, and mapped reads of each replicate. The calculations depicted in the figure have been done using the Binary Alignment Map (BAM) files generated at the marked duplicates stage in the pipelines.

**Fig. 3:**
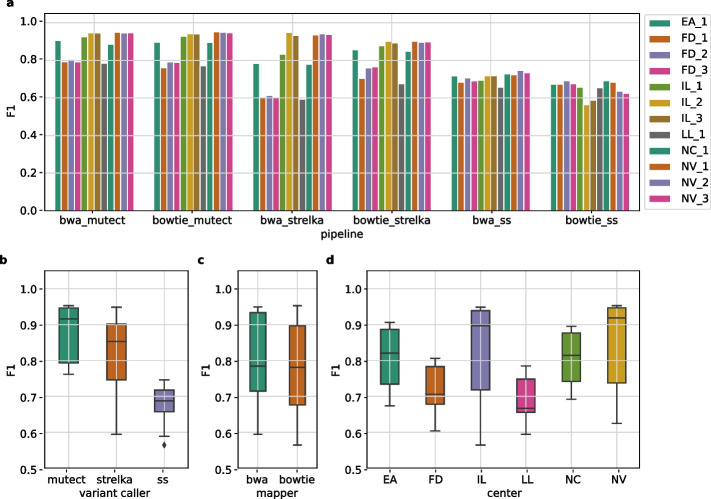
F1 scores and distributions of pipelines in replicates. High-confidence exome SNVs were used as ground truth in the calculations. **a** F1 scores of six pipelines for each replicate. X-axis shows the mapper and variant caller algorithms used in the somatic pipeline, separated by underscores. **b** Distribution of F1 scores according to variant callers. **c** Distribution of F1 scores according to mappers. **d** Distribution of F1 scores according to centers

For each paired tumor-normal FASTQ file, we ran six pipelines (two mappers and three variant callers), resulting in a total of 72 pipelines across the 12 replicates in six centers. Each pipeline’s F1 score was calculated based on the declared high-confidence variants in high-confidence regions, as shown in Fig. 3. Genomic regions (exome and high-confidence) and high-confidence variants were taken from the SEQC2 FTP site [26]. As anticipated, the centers with higher coverage (NV and IL) exhibited better performance, except for *SomaticSniper*. Although the impact of the mapper is not evident in Fig. 3, the influence of variant callers is noticeable. In general, the F1 scores of the variant callers rank in the following order: *Mutect2* > *Strelka2* > *SomaticSniper*. However, there are exceptions that disrupt this trend. For example, the lowest F1 scores were observed using the *bwa_strelka* pipeline (mapper is bwa, variant caller is Strelka2) on FD and LL data.

### Multiply-detected variants of within-center replicates

Previous research has shown that both consensus and ML approaches have the potential to enhance the outcomes of individual pipelines [25, 27, 28]. These studies used a single mapper, and the primary goal was the utilization of different variant callers.

The replicate-based consensus approaches were applied to centers with three biological replicates (FD, IL, and NV), since other centers only had one biological replicate each. In within-center approach, the number of times a variant is detected in the replicates of that center is considered for each pipeline. Multiply detected variants in the replicates from FD (FD_1, FD_2, FD_3), IL (IL_1, IL_2, IL_3), and NV (NV_1, NV_2, NV_3) have been examined. Three possible cases were considered: a variant being detected at least once $\left( {m \ge 1} \right),$ being detected in at least two out of three replicates ($m \ge 2$), and being detected in all three replicates ($m \ge 3$), where “*m*” is defined as the corresponding number of detections.

**Fig. 4:**
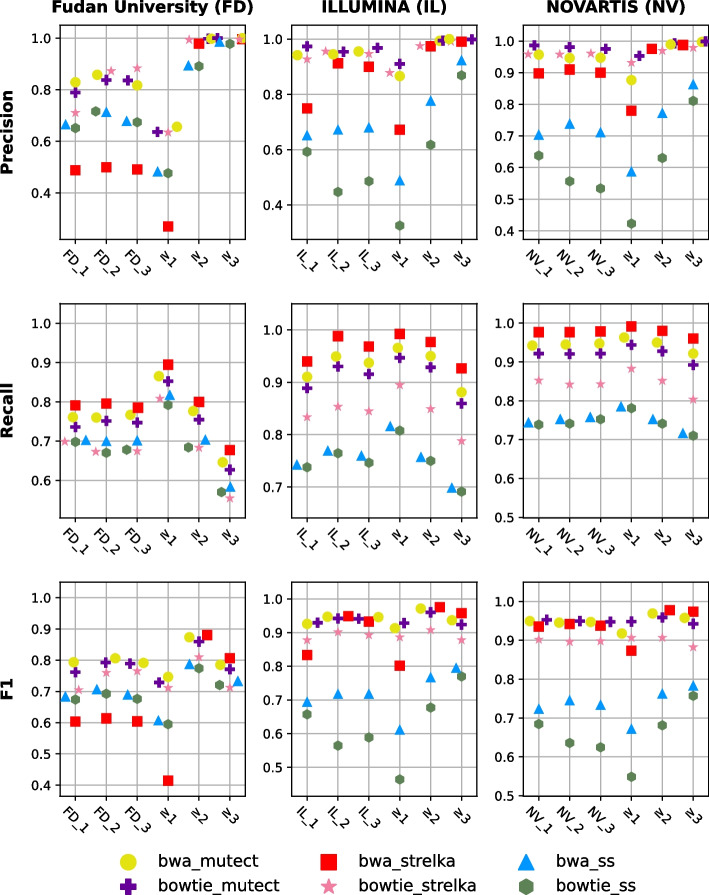
Performance scores of individual replicates and consensus cases of the within-center approach. Calculation of multiple detection cases is based on the number of times variants are detected in the same pipeline replicates within the same center. The first three values on X-axis represent the biological replicates of the corresponding center, while the next three values represent the multiple detection cases. Markers represent the pipelines used

For FD samples, true positive (TP), false positive (FP), false negative (FN), precision, recall, and F1 scores are shown in Table 1. As *m* increases, precision increases, and recall decreases, which is consistent with the expectations (illustrated in Table 1 and Fig. 4). For FD scenarios, the best F1 scores were obtained with two or more replicates ($m \ge 2$), where precision and recall are balanced. Similarly, for IL and NV, the best F1 score for *bwa_mutect*, *bowtie_mutect*, *bwa_strelka,* and *bowtie_strelka* pipelines was obtained by using the variants detected in two or more replicates ($m \ge 2$). In these centers, *SomaticSniper* (*ss*) pipelines achieved the best results with the variants detected in all replicate ($m \ge 3$). Variants detected in two or more replicates consistently exhibited superior F1 score compared to individual replicates (e.g., FD_2) across all centers and pipelines.

**Table 1:** Performance scores of the within-center approach for center replicates of FD

	TP	FP	FN	Precision	Recall	F1
FD_bwa_mutect_m ≥ 1	1003	525	156	0.656	0.865	0.747
FD_bwa_mutect_m ≥ 2	900	2	259	0.998	0.777	0.873
FD_bwa_mutect_m ≥ 3	749	0	410	1.000	0.646	0.785
FD_bowtie_mutect_m ≥ 1	988	565	171	0.636	0.852	0.729
FD_bowtie_mutect_m ≥ 2	875	2	284	0.998	0.755	0.860
FD_bowtie_mutect_m ≥ 3	727	0	432	1.000	0.627	0.771
FD_bwa_strelka_m ≥ 1	1037	2808	122	0.270	0.895	0.414
FD_bwa_strelka_m ≥ 2	927	20	232	0.979	0.800	0.880
FD_bwa_strelka_m ≥ 3	785	3	374	0.996	0.677	0.806
FD_bowtie_strelka_m ≥ 1	937	539	222	0.635	0.808	0.711
FD_bowtie_strelka_m ≥ 2	792	5	367	0.994	0.683	0.810
FD_bowtie_strelka_m ≥ 3	643	3	516	0.995	0.555	0.712
FD_bwa_ss_m ≥ 1	948	1013	211	0.483	0.818	0.608
FD_bwa_ss_m ≥ 2	816	96	343	0.895	0.704	0.788
FD_bwa_ss_m ≥ 3	677	9	482	0.987	0.584	0.734
FD_bowtie_ss_m ≥ 1	918	1009	241	0.476	0.792	0.595
FD_bowtie_ss_m ≥ 2	793	97	366	0.891	0.684	0.774
FD_bowtie_ss_m ≥ 3	661	15	498	0.978	0.570	0.720

The notation "m" is used for multiple detection results. It indicates how many times a variant has been captured in pipelines generated using data from FD replicates. For example, "FD_bwa_mutect_m ≥ 2" indicates the scores of the variants detected in two or more bwa_mutect pipelines in FD replicates

Additionally, to assess the performance of having only two replicates; we performed pairwise combination (intersection) analyses for the within-center approach using the same replicates (Additional file 1: Fig. S3). This investigation focused on identifying somatic variants that were concurrently detected in pairwise subsets of the triple replicates. The F1 scores of the pairwise combinations and the consensus approach for $m \ge 2$ cases are similar in the within-center replicates. However, the F1 scores of the FD center's consensus approach for $m \ge 2$ cases are notably higher than the pairwise F1 score of the same center. Consensus approaches use variants that are detected by all the replicates, as well as variants that are detected by the majority of the replicates. This consensus prediction power is anticipated to be more effective, especially for replicates less than 100× (FD).

### Multiply-detected variants of cross-center replicates

To measure the potential gain from the replicates from different centers, replicates with the same replicate number were combined. Comparison groups were defined as the first replicates (FD_1, IL_1, NV_1), the second replicates (FD_2, IL_2, NV_2), and the third replicates (FD_3, IL_3, NV_3). As it would be easier for clarity, we combined replicates with the same number.

**Fig. 5:**
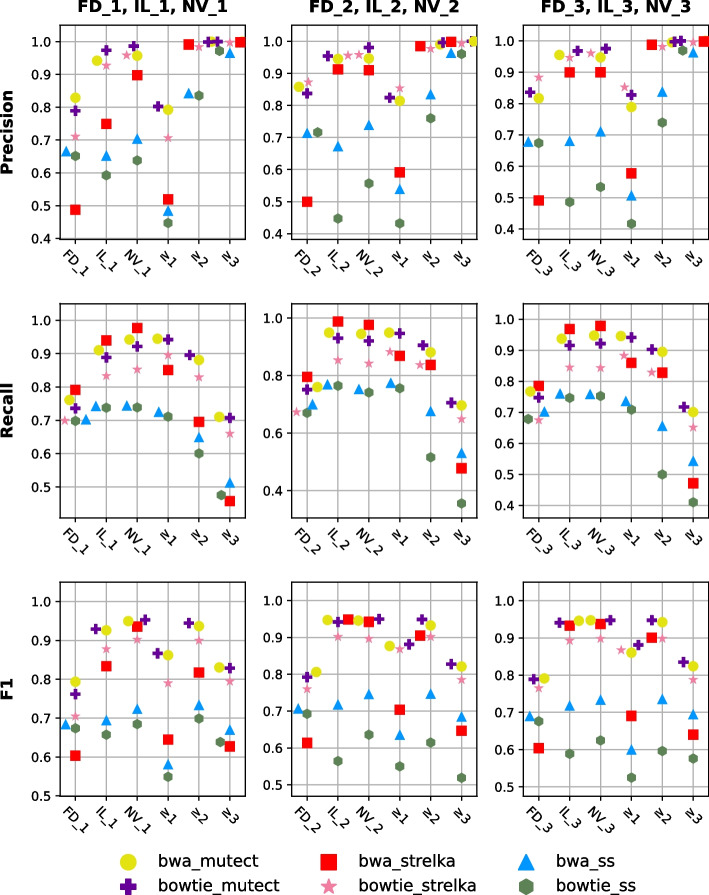
Performance scores of individual replicates and consensus cases of the cross-center approach. Calculation of multiple detection cases based on the detection of variants in the same pipeline replicates with the identical numbers across different centers. The first three values on X-axis represent the biological replicates of the corresponding center, while the next three values represent the multiple detection cases. Markers represent the pipelines used

Figure 5 displays the F1 scores for each pipeline, focusing on the first, second, and third replicates, as well as the individual replicates, considering various multiple detection scenarios. The variants detected in two or more cases ($m \ge 2$) have achieved the highest performance among all multiple detection cases. However, in some pipelines, the performance is only slightly inferior to the best individual result. Interestingly, the replicates from the same center (previous section) has a better performance compared to the approach where replicates from different centers are combined.

We also conducted analyses of pairwise combinations (intersections) across centers using the same replicates (Additional file 1: Fig. S4). In this case, we analyzed pairwise subsets of triple replicates that shared the same ID number. Recall scores for the subset containing FD were lower compared with the remaining subsets. Therefore, F1 scores for the FD subset were found to be low.

### Multiply-detected variants of all replicates

**Fig. 6:**
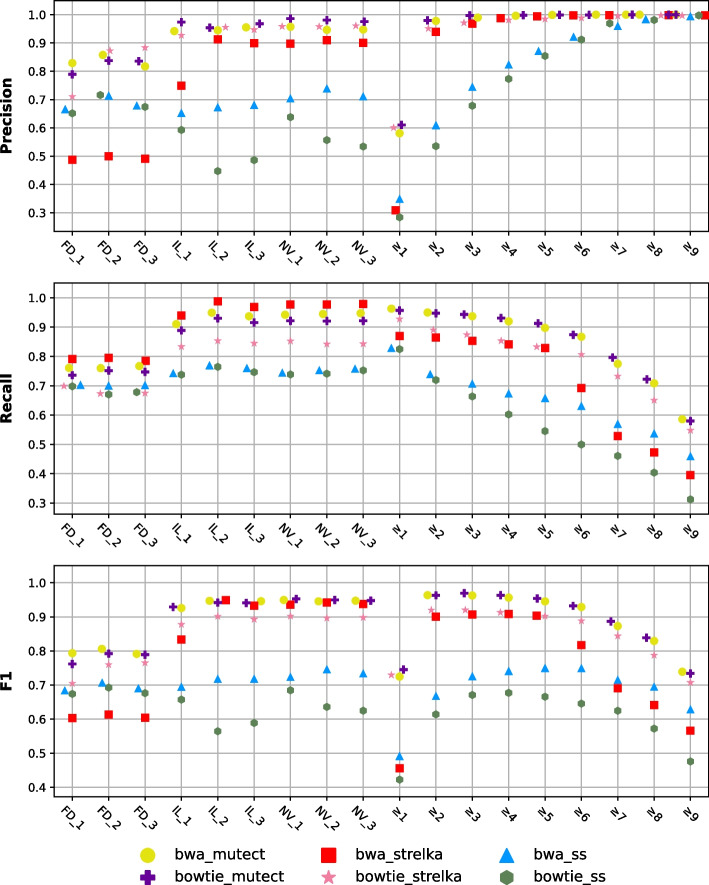
Performance scores of individual replicates and consensus cases of the all centers approach. Calculation of multiple detection cases is based on the number of times variants are detected in the same pipeline replicates in all centers. The first nine values on X-axis represent the biological replicates of the corresponding center, while the next nine values represent the multiple detection cases. Markers represent the pipelines used

By merging within- and cross-center replicates, a total of nine scenarios (3 × 3) were created to evaluate the overall effect of large replicate counts. F1 scores of the pipelines created using all replicates from all centers, along with the individual pipelines, are shown in Fig. 6. It has been clearly observed that as the required number of multiply-detected variants increases, precision increases while recall decreases. Optimal precision-recall balance is achieved around three detections. Compared to the alternative methods mentioned in previous sections (within-center and cross-center), the F1 score performance of this method lies in between. When all approaches are compared, the best result is obtained with the “variants detected in two or more replicates ($m \ge 2$) within-center” approach.

### Machine learning models labeled with multiply-detected variants in the replicates

There are very few sequencing datasets that include declared ground truth (gt) somatic variants. This situation makes it difficult to develop machine learning models as labeled data is required during the training phase, and training-test sets should be similar for good performance. To address this problem, we trained NeuSomatic [25] machine learning models based on the results of multiply-detected approaches. NeuSomatic machine learning models have been developed in ensemble mode with default parameters. We chose the ensemble mode, which leverages results from other variant callers, because initial experiments conducted with the standalone mode yielded relatively low performance (with an F1 score of 0.7).

The input to the NeuSomatic Ensemble models were the features of the first replicate and declared high-confidence region files. The features of the first replicate are recalibrated bwa Tumor/Normal BAM files and Variant Call Format (VCF) files (*bwa_mutect, bwa_strelka, bwa_ss*). The training set uses the data in the first five chromosome regions, and the test set uses the data in the remaining chromosome regions. Since the dataset consists of biological replicates of one sample, chromosome region separation was performed in this manner to avoid overfitting. When training these models, the results of the multiply-detected approaches and declared high-confidence variants were used for labeling. By accepting high-confidence variants as ground truth, the performance of the trained models was measured on the test set.

**Fig. 7:**
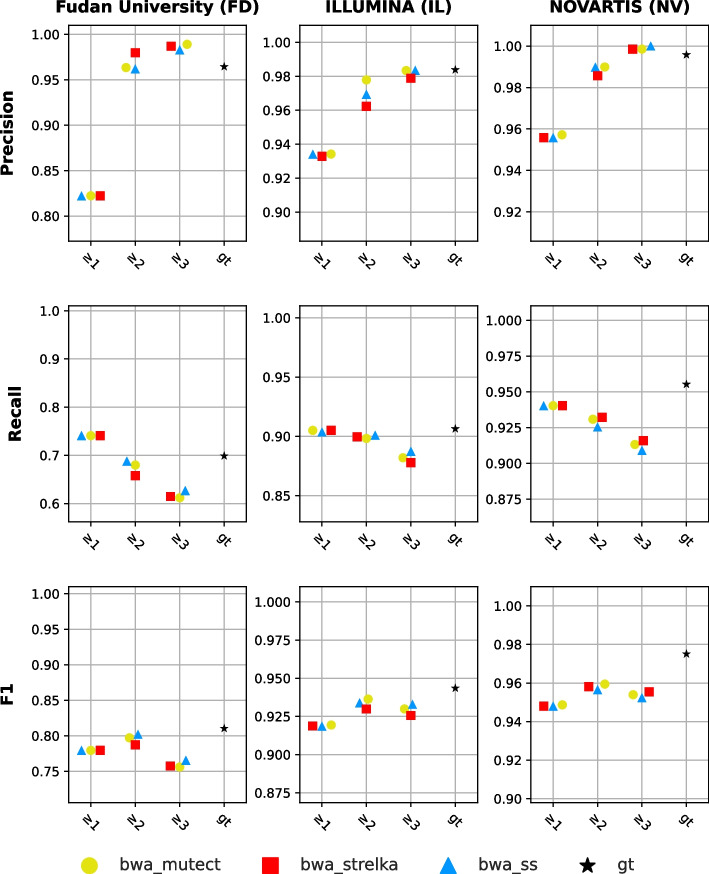
Performance scores of NeuSomatic Ensemble models labeled with within-center approach consensus results and ground truth (gt). Markers indicate which pipeline results are used in the multiple detection cases. X-axis indicates which labeled data is used in training. "gt" indicates ground truth labeled-data is used, and the numbers indicate which multiple detected variants are being used in training

For center-specific experiments, within-center approach consensus results were used for labeling in NeuSomatic Ensemble models in the training stage. The within-center approach was applied to centers with three biological replicates. Therefore, each center-specific experiment used three biological replicates for labeling. Recalibrated bwa BAM files, generated from the first replicate of each center, were used as a BAM input in the training and test sets. For example, the FD_1 bwa BAM file was used as an input in NeuSomatic Ensemble models for FD center data. Since NeuSomatic models were trained in ensemble mode, variant caller VCF files are also required as input. VCF files (*bwa_mutect*, *bwa_strelka*, and *bwa_ss*) of the first replicates (i.e., FD_1) were used as input to NeuSomatic Ensemble models. The performance scores of NeuSomatic Ensemble models trained with within-center approach consensus results and declared high-confidence (gt) variants are as shown in Fig. 7. It has been observed in NeuSomatic Ensemble models; as the required number of detected variants increases, the precision decreases, and the recall increases, similar to the consensus approaches. Especially, NeuSomatic Ensemble models trained by training sets labeled with two or more detected variants ($m \ge 2$) have provided results close to the ground truth-trained model.

**Fig. 8:**
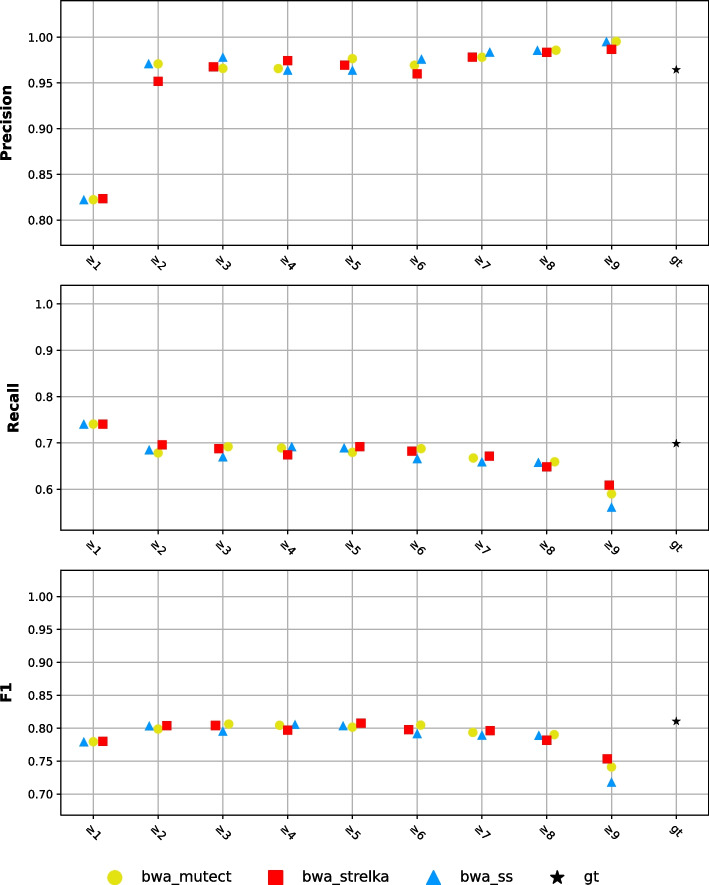
Performance scores of NeuSomatic Ensemble models labeled all centers approach consensus results and ground truth (gt). Markers indicate which pipeline results are used in the multiple detection cases. X-axis indicates which labeled data is used in training. "gt" indicates ground truth labeled-data is used, and the numbers indicate which multiple detected variants are being used in training

For all replicates experiments, all nine biological replicates were used for labeling in NeuSomatic Ensemble models. Recalibrated FD_1 bwa BAM files were employed as the input for all the training and test sets. Additionally, *bwa_mutect*, *bwa_strelka*, and *bwa_ss* VCF files of FD_1 were given as the input for the ensemble machine learning models. The performance scores of NeuSomatic Ensemble models trained with multiply-detected variants and ground truth (gt) are shown in Fig. 8. The outcomes imply that as the required number of variant detections increases, precision increases and recall decreases. However, it should be noted that this pattern was not consistently observed, especially in $m \ge 5$ and $m \ge 6$ cases. The reason for this exception may be due to the fact that for these cases, the number of true positives does not vary significantly compared to the alternatives. The model trained with multiply-detected variants and the one trained with the ground truth performed similarly in some cases.

Machine learning models trained using replicate-based SomaticSniper pipeline results generally yielded better results than SomaticSniper's replicate-based consensus results (Figs. 4, 5 and 6 vs. Figs. 7 and 8). In fact, they achieved results similar to those trained using Mutect2 and Strelka2 pipeline results. This may be because machine learning models utilize Mutect2 and Strelka2 results as features in the training stage. Furthermore, ML models trained with replicate-based consensus approaches performed similarly to those trained with declared high-confidence variants.

## Discussion

In this work, we used the SEQC2-WES dataset, which is based on non-tumor and tumor cell lines from a single patient [15]. Using only a single tumor is a limitation for us and for many benchmarking studies. Unfortunately, obtaining detailed profiling for even a few samples is extremely expensive. Typically, it involves a combination of long reads and deep sequencing. As an alternative, less demanding approach, the proposed replicate-based consensus variant calling has achieved similar results to the ground truth for machine learning training.

We focused on the exome sequencing of somatic samples, considering the wide use of WES in tumor sequencing [29]. For clinical WES, coverage is known to be an important predictor of variant calling performance [30]. The relatively lower success of FD pipelines is likely due to the low coverage of the FD samples. Interestingly, IL-2 replicate had similar performance to NV replicates, despite having significantly higher coverage than them. Our results suggest that obtaining multiple replicates with moderate coverage, such as NV replicates, instead of obtaining a single high-coverage sample like IL_2, may yield superior performance.

In the future, we plan to extend our analyses to germline and whole genome benchmarking datasets to check the validity of findings across a broader spectrum. In our analysis, we focused on declared high-confidence regions to be able to make legitimate comparisons with the declared high-confidence variants. We also did not differentiate regions in DNA; in the future, we plan to consider mapping quality, repetition level, and base quality as additional analyses. In our ML models, we focused on ideal models where profiles and labels are obtained from the same or similar samples to see the maximum gain potential using replicates. Trying distant (unrelated) training-test profiles for more realistic scenarios is an important next step we consider.

## Conclusions

Recent sequencing technologies offer rapid and inexpensive identification of genomic profiles [1]. This leads to their wide usage in research and clinics. Variant identification is a non-trivial and error-prone task, and establishing the reliability of detected variants is critical for the efficient use of these technologies [12]. Sequencing of replicates or related variants (such as multi-region sequencing in tumors) is a common practice [31]. In this work, we have studied a potential variant detection improvement by using multiply-detected variants in replicates. We demonstrated that; via consensus approaches, precision and recall can be substantially improved by using replicates. More importantly, multiply-detected variants can be used to train highly predictive ML models.

## Methods

### SEQC2 dataset for benchmarking somatic mutation calling

SEQC2 dataset includes samples from both Whole Exome Sequencing (WES) and Whole Genome Sequencing (WGS). It was primarily generated by sequencing the tumor-normal samples, which are triple-negative breast cancer (TNBC) cell line (HCC1395) and a B lymphocyte-derived normal cell line (HCC1395BL) from the same donor from the American Type Culture Collection (ATCC). This dataset contains multiple biological replicates prepared in different centers with different libraries. To detect somatic variants, results from machine learning models, long-read sequencing and high coverage data sequencing were analyzed. Detected variants were classified into four confidence levels: HighConf, MedConf, LowConf, and Unclassified. All detected somatic variants have been shared according to their confidence level for WGS in VCF format [26]. Since we used WES data in our study, this VCF file was BED-processed using the exome target BED file [32] by vcftools [33]. Genome Analysis Toolkit (GATK) CallableLoci was used to identify callable regions with “8 × the average coverage for each sample”, and “minimum mapping quality is 20” parameters. Additionally, this VCF file was also BED-processed with the high-confidence region file [34]. After these filtering operations, a total of 1159 high-confidence somatic single nucleotide variants (SNVs) remained for high-confidence exome regions for our evaluations.

Exome data from the SEQC2 dataset was used in this study, consisting of 12 replicates. These replicates were sequenced using two mappers and three variant callers. Venn diagrams of the resulting pipelines were generated to assess their similarities and differences (Additional file 1: Fig. S2). It was observed that the percentage of variants commonly identified by all pipelines ranged approximately from 33 to 53%. The percentage of variants uniquely detected by a single pipeline varied between approximately 1.77–46.75%. Furthermore, the number and ratio of common and uniquely detected variants by the pipelines showed variations from one replicate to another.

### NGS somatic sequencing

The tools and their versions used for processing NGS data are as shown in Table 2.

**Table 2:** Used tools and their versions in NGS somatic pipelines

Tool and algorithm	Version
Trimmomatic	0.3
Reference Genome	GRCh38.d1.vd1
bwa	0.7.15
bowtie2	2.3.5.1
SAMtools	1.8
Picard	2.17.11
Qualimap	2.2.2a
GATK4	4.3.0.0
Strelka2	2.9.10
SomaticSniper	1.0.5.0
vcftools	0.1.16
bcftools	1.9
PyVCF	0.6.8
NeuSomatic	0.2.1
SomaticSeq	2.7.2

Trimmomatic was used for trimming. bwa and bowtie2 were used in mapping. SAMtools were used for converting SAM to BAM, indexing, and sorting. Picard was used to add readgroups and mark duplicates. The GATK4 tools used were BaseRecalibrator, ApplyBQSR, Mutect2, and SelectVariants. NeuSomatic and SomaticSeq were used for machine learning

#### Trimming

Low-quality base reads and adapters were trimmed using Trimmomatic [35]with PE options. Trimmomatic runs with the following parameters in 24 threads:

-phred 33 ILLUMINACLIP: TruSeq3-PE.fa:2:36:10 LEADING:10 TRAILING:10 MAXINFO:50:0.97 MINLEN:20

“TruSeq3-PE.fa” file can be accessed from the “adapters” folder inside the Trimmomatic directory.

#### Mapping

After trimming the reads, they were mapped to the reference genome using bwa and bowtie 2 with 24 threads. Instead of using pre-built index files for the mapping algorithms, the tools were also run in index mode using the reference genome (GRCh38.d1.vd1.fa) file to create the index files. Additionally, bwa mem runs with the “-M” option, and for bowtie 2, the “-x” option is used along with “-1” and “-2” to specify the trimmed_fastq files to be used.

#### Pre-processing

Sequence Alignment Map (SAM) files generated from the mapping stage were converted to BAM format using SAMtools [36] view with “-bS” parameters. SAMtools were also used for indexing and sorting with default parameters.

Subsequently, read group information added using Picard with the parameters “CREATE_INDEX = True VERBOSITY = INFO QUIET = false VALIDATION_STRINGENCY = LENIENT COMPRESSION_LEVEL = 5” to the generated BAM files. Afterwards, Picard MarkDuplicates was executed with the parameters “CREATE_INDEX = true ASSUME_SORTED = true MAX_FILE_HANDLES_FOR_READ_ENDS_MAP = 1000 VALIDATION_STRINGENCY = LENIENT”.

Finally, GATK's [37] BaseRecalibrator and ApplyBQSR tools were run to the generated BAM files sequentially. In BaseRecalibrator, three --known-sites arguments were used (db_snp file (hg38_v0_Homo_sapiens_assembly38.dbsnp138.vcf), Mills and 1000G gold standard file (Mills_and_1000G_gold_standard.indels.hg38.vcf.gz) and 1000G phase1 snps file (1000G_phase1.snps.high_confidence.hg38.vcf.gz)). These files can be obtained from the GATK resource bundle [38]. Lastly, GATK's ApplyBQSR tool was run with--bqsr-recal-file argument (generated in the BaseRecalibrator step).

#### Variant calling

After creating the tumor-normal recalibrated BAM files, three variant callers were executed. These variant callers were Mutect2, Strelka2, and SomaticSniper. If there was a ploidy issue in the resulting variant caller files, it was corrected using bcftools fixploidy tool. Additionally, the outputs of all variant callers were BED-processed using vcftools with the high-confidence region and exome target BED files. This ensured that the variants detected in the exome high-confidence region were used in the calculations.

Mutect2 runs with “-normal” parameters with 24 threads. The detected variants (vcf file) were filtered using GATK's FilterMutectCalls tool. Finally, GATK SelectVariants tool was used to save SNV and indel variants into separate VCF files.

Due to Strelka2 not being compatible with Python 3, a separate Python 2.7 environment was set up for Strelka2. Strelka2 was run using this environment. Firstly, ConfigureStrelkaSomaticWorkflow.py was run with the “--exome” parameter and “runWorkflow.py” script was generated. Then, the generated "runWorkflow.py" script was executed with the "-m local -j 23" parameters.

SomaticSniper was run with the parameters “-F vcf -Q 40 -G -L”. Since SomaticSniper does not have a recommended filtering method for files mapped with bowtie, no filtering was applied to the SomaticSniper vcf files.

While only the PASS variants were selected for Mutect2 and Strelka2, no filtering was applied to SomaticSniper results. When SomaticSniper results were filtered, the number of detected somatic variants decreased significantly, which greatly reduced the F1 score.

### NeuSomatic

While installing NeuSomatic, the first step was to compile it using g++ 5.4.0. During this compilation, there were issues with Seqan library. To resolve this problem, a compatible version of Seqan for gcc 5 was found, and its master branch link was provided in “seqan.cmake” file in “third-party” folder. After the compilation process, Python 3.7 environment was set up, followed by meeting other dependencies. Finally, the functionality of NeuSomatic was verified by running “run_test.sh”. However, during the execution, there was an error related to the “pillow” Python library. To fix this issue, pillow version was downgraded to 6.2.1. After successfully passing the test, NeuSomatic was executed in CPU mode, and machine learning models were developed.

For creating NeuSomatic models, the first five chromosomes were used for training, and the remaining chromosomes were used for testing. The recalibrated BAM file was divided into chromosome-based regions using the “samtools view” tool. The resulting training and test BAM files were re-indexed and sorted. Region files were manually split. For creating the ground truth VCF file, variants were first converted to a pandas DataFrame and then saved as a VCF file using the “to_csv()” function. Subsequently, the necessary VCF headers were added to this VCF file.

An Ensemble.tsv file containing the features is required to train NeuSomatic Ensemble models. This Ensemble.tsv file was generated using SomaticSeq.Wrapper.sh in SomaticSeq [39]. The resulting Ensemble.sSNV.tsv file had some “None” values, which were replaced with 0. Additionally, in some cases, the first column in this tsv file could be "id" instead of "CHROM". When the first column was “id”, NeuSomatic Ensemble models did not work properly. Therefore, in cases where the first column was labeled as "id," after dropping that column, it was ensured that the first column became "CHROM". The tsv file and other training files were used as input to run the NeuSomatic preprocess.py with the parameters "--mode train --min_mapq 10 --threads 12". After preprocessing, NeuSomatic train.py was executed with the parameters "--ensemble --batch size 100 --num_threads 12".

Similarly, for the test files, NeuSomatic preprocess.py and call.py were executed. Finally, SNVs were extracted from the detected variants in "Neusomatic.vcf" file using GATK SelectVariants.

### Evaluation metrics

The number of detected somatic variants remains very low when considering the total base count in the DNA. This also leads to somatic variant sets being unbalanced. Therefore, the metrics commonly used in unbalanced datasets in Table 3 were employed to assess the performance of the models. During the calculations, the VCF file operations were performed using PyVCF [40]. While determining the truth of the detected variants, it was checked whether the CHROM POS REF and ID information were the same as the high-confidence variants.

**Table 3:** Metrics used to measure the performance of models detecting somatic variants

Metric	Descriptions
TP	The number of correctly predicted somatic variants
FP	The number of predicted somatic variants that are not actually somatic variants
FN	The number of actual somatic variants that were incorrectly classified as non-somatic (missed) by the model
Precision	$\frac{TP}{{TP + FP}}$
Recall	$\frac{TP}{{TP + FN}}$
F1	$\frac{2 \times Precision \times Recall}{{Precision + Recall}}$

### Supplementary Information

**Additional file 1:** Contains detailed performance scores of all pipelines, approaches, and machine learning methods used in the study in Fig. S1 and Tables S1–S7. It also contains Venn diagrams of the somatic variants detected by the pipelines (Fig. S2) and the results of the pairwise analyses (Figs. S3 & S4).

## Data Availability

All data analyzed during this study are included in this published article [15]. All the raw sequencing data (FASTQ files) can be accessed from NCBI's Sequence Read Archive (SRP162370). The region (bed) files and the high-confidence variants in the vcf file used in this study are available at “https://ftp-trace.ncbi.nlm.nih.gov/ReferenceSamples/seqc/Somatic_Mutation_WG”. Sequence Read Archive links of each of the tumor-normal replicates sequenced in the centers can be accessed from the website “https://sites.google.com/view/seqc2/”.

## Abbreviations

ATCC: American Type Culture Collection
BAM: Binary alignment map
EA: European Infrastructure for Translational Medicine
FD: Fudan University
FN: False negative
FP: False positive
GATK: Genome analysis toolkit
gt: Ground truth
IL: Illumina
LL: Loma Linda University
m: The required number of detections
ML: Machine learning
NC: National Cancer Institute
NGS: Next generation sequencing
NV: Novartis
SAM: Sequence alignment map
SEQC2: Sequencing quality control phase 2
SNV: Single nucleotide variant
ss: Somatic Sniper
TNBC: Triple-negative breast cancer
TP: True positive
VCF: Variant call format
WES: Whole exome sequencing
WGS: Whole genome sequencing
