# Correction: Improving somatic exome sequencing performance by biological replicates

**DOI:** 10.1186/s12859-024-05828-0

**Published:** 2024-06-25

**Authors:** Yunus Emre Cebeci, Rumeysa Aslihan Erturk, Mehmet Arif Ergun, Mehmet Baysan

**Affiliations:** Department of Computer Engineering, Istanbul Technical University, 34469 Istanbul, Turkey

**Correction to: BMC Bioinformatics (2024) 25:124** 10.1186/s12859-024-05742-5

Following the publication of the original article [1], the authors identified that the funding information was missing from the funding statement. The correct funding is given below.

The correct funding is: This study is partially funded by Istanbul Technical University (ITU) Scientific Research Projects Office (Project Title: Cancer Sequencing Algorithms Performance Comparisons, Grant Number: 43239).

The original article [1] has been corrected.
