# Supplementary material for: Improving somatic exome sequencing performance by biological replicates
Source: BMC Bioinformatics. 2024 Mar 22;25:124. doi: 10.1186/s12859-024-05742-5 (PMC10958848; doi:10.1186/s12859-024-05742-5)
Supplement: Supplementary file 1 — Additional file 1: Contains detailed performance scores of all pipelines, approaches, and machine learning methods used in the study in Fig. S1 and Tables S1–S7. It also contains Venn diagrams of the somatic variants detected by the pipelines (Fig. S2) and the results of the pairwise analyses (Figs. S3 & S4). [file 12859_2024_5742_MOESM1_ESM.docx]

**
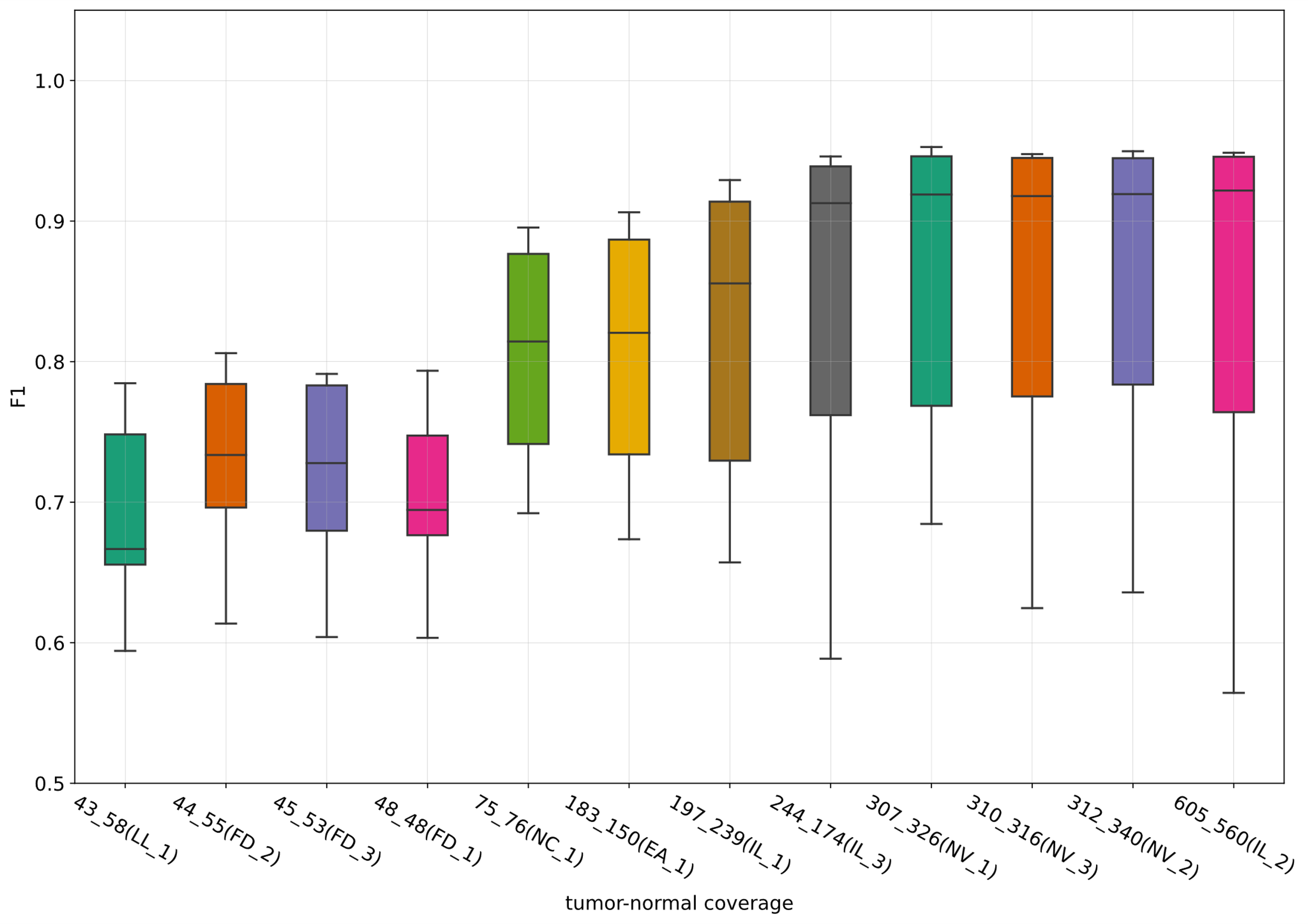
**

**Figure S1. F1 score distributions of pipelines according to tumor-normal coverage.** X-axis, separated by an underscore and bracket, shows the tumor coverage, normal coverage, replicate name, and replicate number of each replicate, respectively. Y-axis shows the distribution of F1 scores.

**
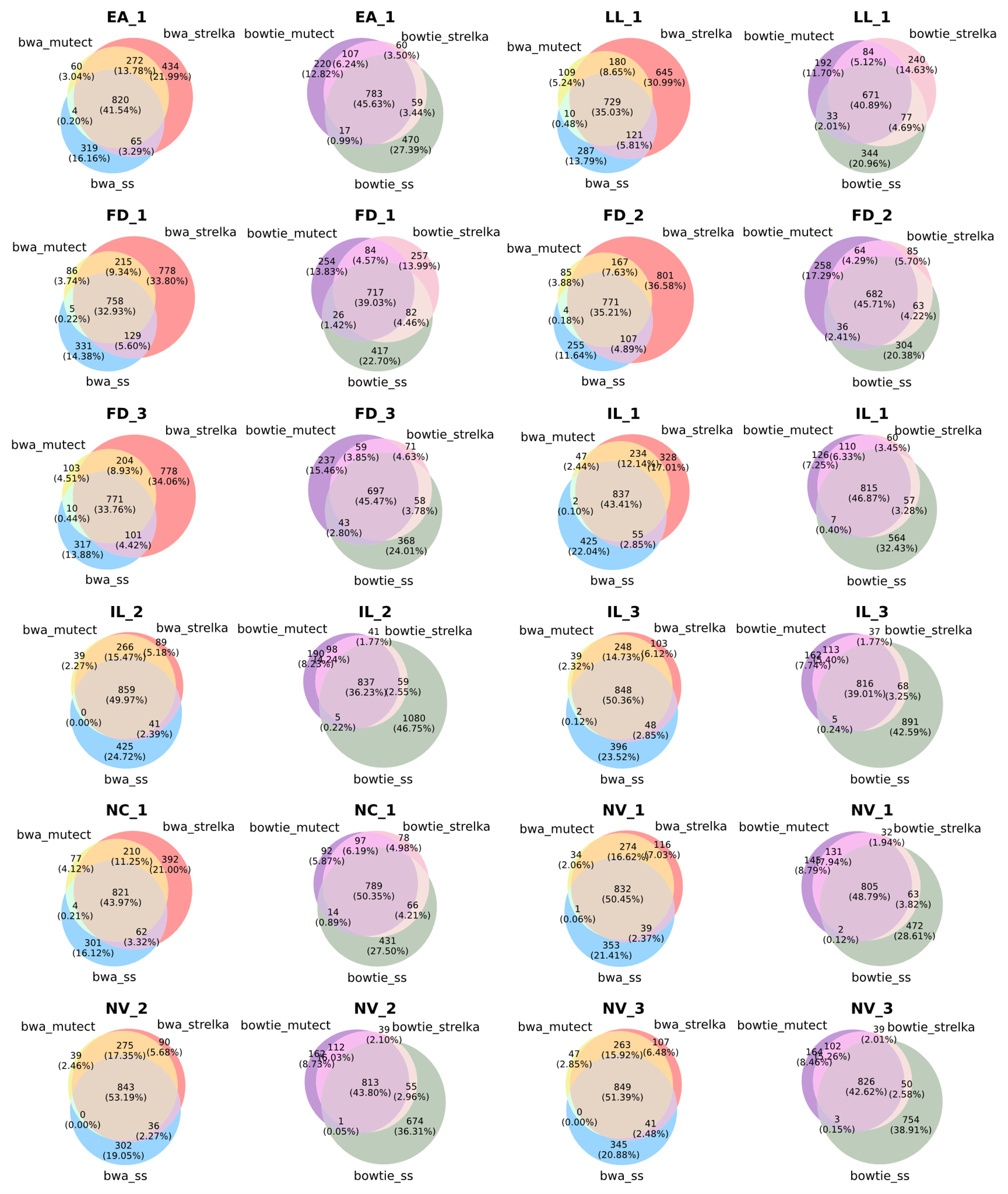
**

**Figure S2. Venn diagrams of variants detected by pipelines in each replicate, with bwa and bowtie separate.**

**
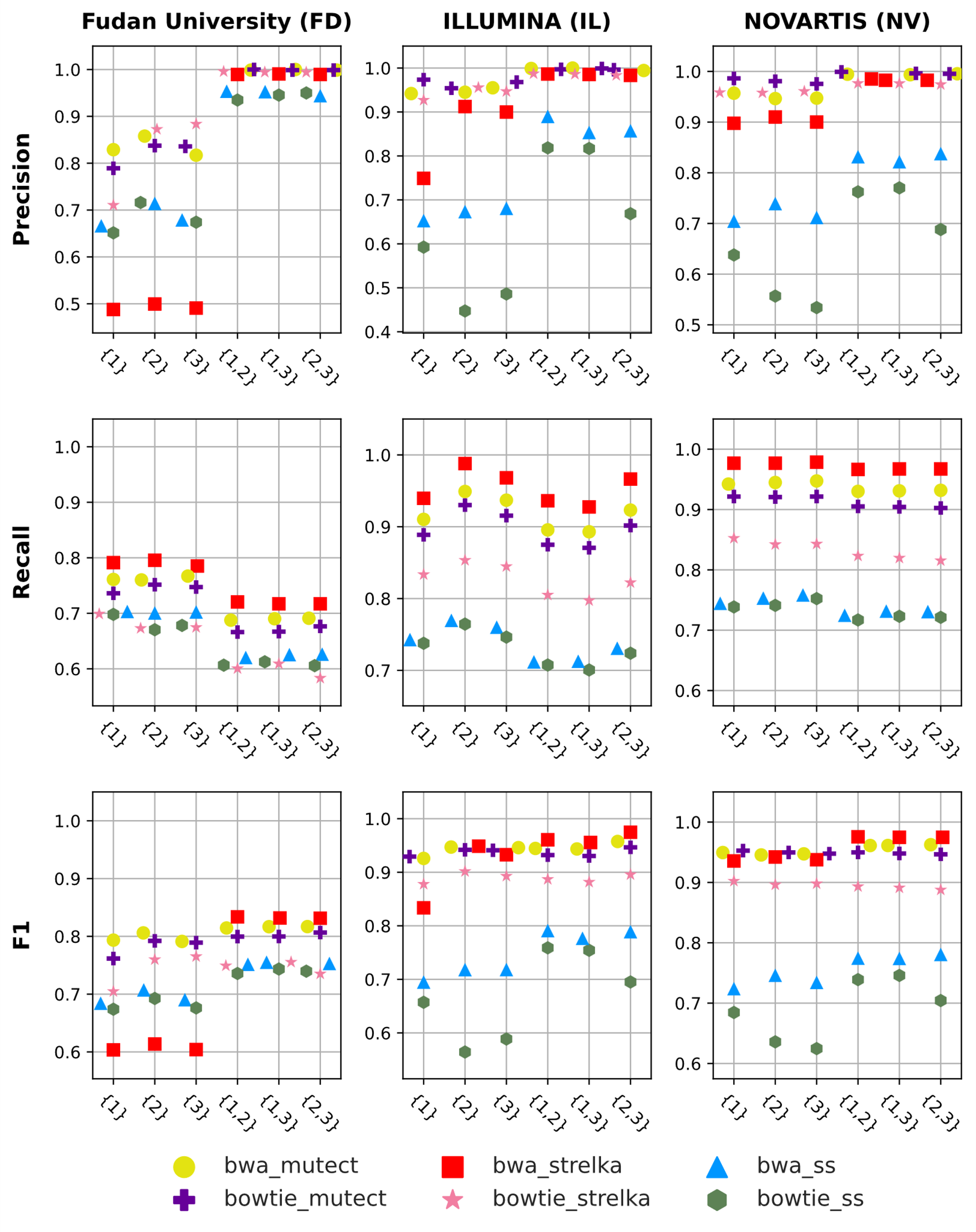
**

**Figure S3.** **Performance scores of individual and pairwise combinations of replicates in the within-center approach.** The first three values on the X-axis represent biological replicates in the corresponding center, while the next three values represent pairwise combinations (subsets) of replicates. The calculation of pairwise combinations is based on the detection of common variants (intersection) between two replicates within the same pipeline within the same center. Markers represent the pipelines used.

**
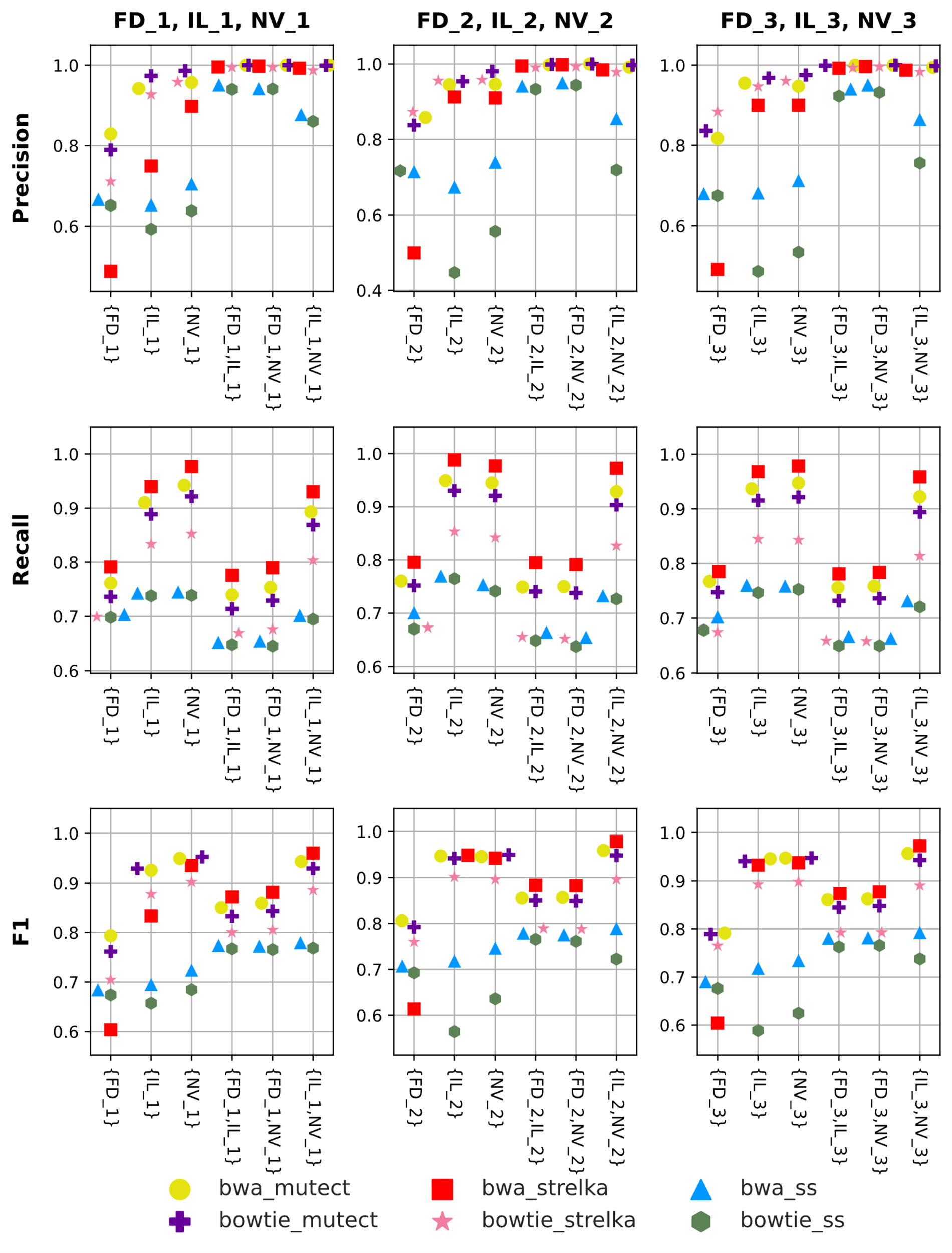
**

**Figure S4. Performance scores of individual and pairwise combinations of replicates in the cross-center approach.** The first three values on the X-axis represent biological replicates in the corresponding center, while the next three values represent pairwise combinations (subsets) of replicates. The calculation of pairwise combinations is based on the detection of common variants (intersection) between two replicates within the same pipeline with identical numbers across different centers. Markers represent the pipelines used.

**Table S1** Performance scores of the pipelines in the replicates.

|  | **TP** | **FP** | **FN** | **Precision** | **Recall** | **F1** |
| --- | --- | --- | --- | --- | --- | --- |
| EA_1_bowtie_mutect | 1025 | 102 | 134 | 0.909 | 0.884 | 0.897 |
| EA_1_bowtie_ss | 838 | 491 | 321 | 0.631 | 0.723 | 0.674 |
| EA_1_bowtie_strelka | 929 | 80 | 230 | 0.921 | 0.802 | 0.857 |
| EA_1_bwa_mutect | 1049 | 107 | 110 | 0.907 | 0.905 | 0.906 |
| EA_1_bwa_ss | 849 | 359 | 310 | 0.703 | 0.733 | 0.717 |
| EA_1_bwa_strelka | 1078 | 513 | 81 | 0.678 | 0.930 | 0.784 |
| FD_1_bowtie_mutect | 853 | 228 | 306 | 0.789 | 0.736 | 0.762 |
| FD_1_bowtie_ss | 809 | 433 | 350 | 0.651 | 0.698 | 0.674 |
| FD_1_bowtie_strelka | 810 | 330 | 349 | 0.711 | 0.699 | 0.705 |
| FD_1_bwa_mutect | 882 | 182 | 277 | 0.829 | 0.761 | 0.794 |
| FD_1_bwa_ss | 815 | 408 | 344 | 0.666 | 0.703 | 0.684 |
| FD_1_bwa_strelka | 917 | 963 | 242 | 0.488 | 0.791 | 0.603 |
| FD_2_bowtie_mutect | 871 | 169 | 288 | 0.838 | 0.752 | 0.792 |
| FD_2_bowtie_ss | 777 | 308 | 382 | 0.716 | 0.670 | 0.693 |
| FD_2_bowtie_strelka | 780 | 114 | 379 | 0.872 | 0.673 | 0.760 |
| FD_2_bwa_mutect | 881 | 146 | 278 | 0.858 | 0.760 | 0.806 |
| FD_2_bwa_ss | 812 | 325 | 347 | 0.714 | 0.701 | 0.707 |
| FD_2_bwa_strelka | 922 | 924 | 237 | 0.499 | 0.796 | 0.614 |
| FD_3_bowtie_mutect | 866 | 170 | 293 | 0.836 | 0.747 | 0.789 |
| FD_3_bowtie_ss | 786 | 380 | 373 | 0.674 | 0.678 | 0.676 |
| FD_3_bowtie_strelka | 782 | 103 | 377 | 0.884 | 0.675 | 0.765 |
| FD_3_bwa_mutect | 889 | 199 | 270 | 0.817 | 0.767 | 0.791 |
| FD_3_bwa_ss | 814 | 385 | 345 | 0.679 | 0.702 | 0.690 |
| FD_3_bwa_strelka | 910 | 944 | 249 | 0.491 | 0.785 | 0.604 |
| IL_1_bowtie_mutect | 1030 | 28 | 129 | 0.974 | 0.889 | 0.929 |
| IL_1_bowtie_ss | 855 | 588 | 304 | 0.593 | 0.738 | 0.657 |
| IL_1_bowtie_strelka | 966 | 76 | 193 | 0.927 | 0.833 | 0.878 |
| IL_1_bwa_mutect | 1055 | 65 | 104 | 0.942 | 0.910 | 0.926 |
| IL_1_bwa_ss | 861 | 458 | 298 | 0.653 | 0.743 | 0.695 |
| IL_1_bwa_strelka | 1089 | 365 | 70 | 0.749 | 0.940 | 0.834 |
| IL_2_bowtie_mutect | 1078 | 52 | 81 | 0.954 | 0.930 | 0.942 |
| IL_2_bowtie_ss | 886 | 1095 | 273 | 0.447 | 0.764 | 0.564 |
| IL_2_bowtie_strelka | 989 | 46 | 170 | 0.956 | 0.853 | 0.902 |
| IL_2_bwa_mutect | 1100 | 64 | 59 | 0.945 | 0.949 | 0.947 |
| IL_2_bwa_ss | 892 | 433 | 267 | 0.673 | 0.770 | 0.718 |
| IL_2_bwa_strelka | 1145 | 110 | 14 | 0.912 | 0.988 | 0.949 |
| IL_3_bowtie_mutect | 1061 | 35 | 98 | 0.968 | 0.915 | 0.941 |
| IL_3_bowtie_ss | 865 | 915 | 294 | 0.486 | 0.746 | 0.589 |
| IL_3_bowtie_strelka | 979 | 55 | 180 | 0.947 | 0.845 | 0.893 |
| IL_3_bwa_mutect | 1086 | 51 | 73 | 0.955 | 0.937 | 0.946 |
| IL_3_bwa_ss | 881 | 413 | 278 | 0.681 | 0.760 | 0.718 |
| IL_3_bwa_strelka | 1122 | 125 | 37 | 0.900 | 0.968 | 0.933 |
| LL_1_bowtie_mutect | 826 | 154 | 333 | 0.843 | 0.713 | 0.772 |
| LL_1_bowtie_ss | 748 | 377 | 411 | 0.665 | 0.645 | 0.655 |
| LL_1_bowtie_strelka | 754 | 318 | 405 | 0.703 | 0.651 | 0.676 |
| LL_1_bwa_mutect | 858 | 170 | 301 | 0.835 | 0.740 | 0.785 |
| LL_1_bwa_ss | 758 | 389 | 401 | 0.661 | 0.654 | 0.657 |
| LL_1_bwa_strelka | 842 | 833 | 317 | 0.503 | 0.726 | 0.594 |
| NC_1_bowtie_mutect | 963 | 29 | 196 | 0.971 | 0.831 | 0.895 |
| NC_1_bowtie_ss | 851 | 449 | 308 | 0.655 | 0.734 | 0.692 |
| NC_1_bowtie_strelka | 929 | 101 | 230 | 0.902 | 0.802 | 0.849 |
| NC_1_bwa_mutect | 1006 | 106 | 153 | 0.905 | 0.868 | 0.886 |
| NC_1_bwa_ss | 855 | 333 | 304 | 0.720 | 0.738 | 0.729 |
| NC_1_bwa_strelka | 1031 | 454 | 128 | 0.694 | 0.890 | 0.780 |
| NV_1_bowtie_mutect | 1068 | 15 | 91 | 0.986 | 0.921 | 0.953 |
| NV_1_bowtie_ss | 856 | 486 | 303 | 0.638 | 0.739 | 0.685 |
| NV_1_bowtie_strelka | 988 | 43 | 171 | 0.958 | 0.852 | 0.902 |
| NV_1_bwa_mutect | 1092 | 49 | 67 | 0.957 | 0.942 | 0.950 |
| NV_1_bwa_ss | 863 | 362 | 296 | 0.704 | 0.745 | 0.724 |
| NV_1_bwa_strelka | 1132 | 129 | 27 | 0.898 | 0.977 | 0.936 |
| NV_2_bowtie_mutect | 1067 | 21 | 92 | 0.981 | 0.921 | 0.950 |
| NV_2_bowtie_ss | 859 | 684 | 300 | 0.557 | 0.741 | 0.636 |
| NV_2_bowtie_strelka | 976 | 43 | 183 | 0.958 | 0.842 | 0.896 |
| NV_2_bwa_mutect | 1095 | 62 | 64 | 0.946 | 0.945 | 0.946 |
| NV_2_bwa_ss | 873 | 308 | 286 | 0.739 | 0.753 | 0.746 |
| NV_2_bwa_strelka | 1132 | 112 | 27 | 0.910 | 0.977 | 0.942 |
| NV_3_bowtie_mutect | 1068 | 27 | 91 | 0.975 | 0.921 | 0.948 |
| NV_3_bowtie_ss | 872 | 761 | 287 | 0.534 | 0.752 | 0.625 |
| NV_3_bowtie_strelka | 977 | 40 | 182 | 0.961 | 0.843 | 0.898 |
| NV_3_bwa_mutect | 1098 | 61 | 61 | 0.947 | 0.947 | 0.947 |
| NV_3_bwa_ss | 879 | 356 | 280 | 0.712 | 0.758 | 0.734 |
| NV_3_bwa_strelka | 1134 | 126 | 25 | 0.900 | 0.978 | 0.938 |

**Table S2** Performance scores of the within-center approach for consensus cases. The notation "m" is used for multiple detection results. It indicates how many times a variant has been captured.

|  | **TP** | **FP** | **FN** | **Precision** | **Recall** | **F1** |
| --- | --- | --- | --- | --- | --- | --- |
| FD_bowtie_mutect_m≥1 | 988 | 565 | 171 | 0.636 | 0.852 | 0.729 |
| FD_bowtie_mutect_m≥2 | 875 | 2 | 284 | 0.998 | 0.755 | 0.860 |
| FD_bowtie_mutect_m≥3 | 727 | 0 | 432 | 1.000 | 0.627 | 0.771 |
| FD_bowtie_ss_m≥1 | 918 | 1009 | 241 | 0.476 | 0.792 | 0.595 |
| FD_bowtie_ss_m≥2 | 793 | 97 | 366 | 0.891 | 0.684 | 0.774 |
| FD_bowtie_ss_m≥3 | 661 | 15 | 498 | 0.978 | 0.570 | 0.720 |
| FD_bowtie_strelka_m≥1 | 937 | 539 | 222 | 0.635 | 0.808 | 0.711 |
| FD_bowtie_strelka_m≥2 | 792 | 5 | 367 | 0.994 | 0.683 | 0.810 |
| FD_bowtie_strelka_m≥3 | 643 | 3 | 516 | 0.995 | 0.555 | 0.712 |
| FD_bwa_mutect_m≥1 | 1003 | 525 | 156 | 0.656 | 0.865 | 0.747 |
| FD_bwa_mutect_m≥2 | 900 | 2 | 259 | 0.998 | 0.777 | 0.873 |
| FD_bwa_mutect_m≥3 | 749 | 0 | 410 | 1.000 | 0.646 | 0.785 |
| FD_bwa_ss_m≥1 | 948 | 1013 | 211 | 0.483 | 0.818 | 0.608 |
| FD_bwa_ss_m≥2 | 816 | 96 | 343 | 0.895 | 0.704 | 0.788 |
| FD_bwa_ss_m≥3 | 677 | 9 | 482 | 0.987 | 0.584 | 0.734 |
| FD_bwa_strelka_m≥1 | 1037 | 2808 | 122 | 0.270 | 0.895 | 0.414 |
| FD_bwa_strelka_m≥2 | 927 | 20 | 232 | 0.979 | 0.800 | 0.880 |
| FD_bwa_strelka_m≥3 | 785 | 3 | 374 | 0.996 | 0.677 | 0.806 |
| IL_bowtie_mutect_m≥1 | 1097 | 108 | 62 | 0.910 | 0.947 | 0.928 |
| IL_bowtie_mutect_m≥2 | 1076 | 6 | 83 | 0.994 | 0.928 | 0.960 |
| IL_bowtie_mutect_m≥3 | 996 | 1 | 163 | 0.999 | 0.859 | 0.924 |
| IL_bowtie_ss_m≥1 | 936 | 1939 | 223 | 0.326 | 0.808 | 0.464 |
| IL_bowtie_ss_m≥2 | 869 | 538 | 290 | 0.618 | 0.750 | 0.677 |
| IL_bowtie_ss_m≥3 | 801 | 121 | 358 | 0.869 | 0.691 | 0.770 |
| IL_bowtie_strelka_m≥1 | 1037 | 144 | 122 | 0.878 | 0.895 | 0.886 |
| IL_bowtie_strelka_m≥2 | 984 | 25 | 175 | 0.975 | 0.849 | 0.908 |
| IL_bowtie_strelka_m≥3 | 913 | 8 | 246 | 0.991 | 0.788 | 0.878 |
| IL_bwa_mutect_m≥1 | 1119 | 173 | 40 | 0.866 | 0.965 | 0.913 |
| IL_bwa_mutect_m≥2 | 1101 | 7 | 58 | 0.994 | 0.950 | 0.971 |
| IL_bwa_mutect_m≥3 | 1021 | 0 | 138 | 1.000 | 0.881 | 0.937 |
| IL_bwa_ss_m≥1 | 946 | 986 | 213 | 0.490 | 0.816 | 0.612 |
| IL_bwa_ss_m≥2 | 878 | 251 | 281 | 0.778 | 0.758 | 0.767 |
| IL_bwa_ss_m≥3 | 810 | 67 | 349 | 0.924 | 0.699 | 0.796 |
| IL_bwa_strelka_m≥1 | 1150 | 560 | 9 | 0.673 | 0.992 | 0.802 |
| IL_bwa_strelka_m≥2 | 1132 | 30 | 27 | 0.974 | 0.977 | 0.975 |
| IL_bwa_strelka_m≥3 | 1074 | 10 | 85 | 0.991 | 0.927 | 0.958 |
| NV_bowtie_mutect_m≥1 | 1094 | 54 | 65 | 0.953 | 0.944 | 0.948 |
| NV_bowtie_mutect_m≥2 | 1075 | 8 | 84 | 0.993 | 0.928 | 0.959 |
| NV_bowtie_mutect_m≥3 | 1034 | 1 | 125 | 0.999 | 0.892 | 0.943 |
| NV_bowtie_ss_m≥1 | 905 | 1235 | 254 | 0.423 | 0.781 | 0.549 |
| NV_bowtie_ss_m≥2 | 859 | 504 | 300 | 0.630 | 0.741 | 0.681 |
| NV_bowtie_ss_m≥3 | 823 | 192 | 336 | 0.811 | 0.710 | 0.757 |
|  |  |  |  |  |  |  |
| NV_bowtie_strelka_m≥1 | 1023 | 75 | 136 | 0.932 | 0.883 | 0.907 |
| NV_bowtie_strelka_m≥2 | 987 | 31 | 172 | 0.970 | 0.852 | 0.907 |
| NV_bowtie_strelka_m≥3 | 931 | 20 | 228 | 0.979 | 0.803 | 0.882 |
| NV_bwa_mutect_m≥1 | 1116 | 157 | 43 | 0.877 | 0.963 | 0.918 |
| NV_bwa_mutect_m≥2 | 1101 | 12 | 58 | 0.989 | 0.950 | 0.969 |
| NV_bwa_mutect_m≥3 | 1068 | 3 | 91 | 0.997 | 0.921 | 0.958 |
| NV_bwa_ss_m≥1 | 911 | 639 | 248 | 0.588 | 0.786 | 0.673 |
| NV_bwa_ss_m≥2 | 873 | 256 | 286 | 0.773 | 0.753 | 0.763 |
| NV_bwa_ss_m≥3 | 831 | 131 | 328 | 0.864 | 0.717 | 0.784 |
| NV_bwa_strelka_m≥1 | 1149 | 324 | 10 | 0.780 | 0.991 | 0.873 |
| NV_bwa_strelka_m≥2 | 1136 | 29 | 23 | 0.975 | 0.980 | 0.978 |
| NV_bwa_strelka_m≥3 | 1113 | 14 | 46 | 0.988 | 0.960 | 0.974 |

**Table S3** Performance scores of the cross-center approach for consensus cases. 1s is the number one replication scenario (FD_1, IL_1, NV_1). 2s is the number two replication scenario (FD_2, IL_2, NV_2). 3s is the number three replication scenario (FD_3, IL_3, NV_3). The notation "m" is used for multiple detection results. It indicates how many times a variant has been captured.

|  | **TP** | **FP** | **FN** | **Precision** | **Recall** | **F1** |
| --- | --- | --- | --- | --- | --- | --- |
| 1s_bowtie_mutect_m≥1 | 1092 | 269 | 67 | 0.802 | 0.942 | 0.867 |
| 1s_bowtie_mutect_m≥2 | 1038 | 1 | 121 | 0.999 | 0.896 | 0.944 |
| 1s_bowtie_mutect_m≥3 | 820 | 0 | 339 | 1.000 | 0.708 | 0.829 |
| 1s_bowtie_ss_m≥1 | 824 | 1019 | 335 | 0.447 | 0.711 | 0.549 |
| 1s_bowtie_ss_m≥2 | 696 | 137 | 463 | 0.836 | 0.601 | 0.699 |
| 1s_bowtie_ss_m≥3 | 551 | 16 | 608 | 0.972 | 0.475 | 0.638 |
| 1s_bowtie_strelka_m≥1 | 1038 | 431 | 121 | 0.707 | 0.896 | 0.790 |
| 1s_bowtie_strelka_m≥2 | 961 | 16 | 198 | 0.984 | 0.829 | 0.900 |
| 1s_bowtie_strelka_m≥3 | 765 | 2 | 394 | 0.997 | 0.660 | 0.794 |
| 1s_bwa_mutect_m≥1 | 1095 | 287 | 64 | 0.792 | 0.945 | 0.862 |
| 1s_bwa_mutect_m≥2 | 1021 | 0 | 138 | 1.000 | 0.881 | 0.937 |
| 1s_bwa_mutect_m≥3 | 823 | 0 | 336 | 1.000 | 0.710 | 0.830 |
| 1s_bwa_ss_m≥1 | 841 | 893 | 318 | 0.485 | 0.726 | 0.581 |
| 1s_bwa_ss_m≥2 | 753 | 140 | 406 | 0.843 | 0.650 | 0.734 |
| 1s_bwa_ss_m≥3 | 595 | 21 | 564 | 0.966 | 0.513 | 0.670 |
| 1s_bwa_strelka_m≥1 | 986 | 913 | 173 | 0.519 | 0.851 | 0.645 |
| 1s_bwa_strelka_m≥2 | 806 | 7 | 353 | 0.991 | 0.695 | 0.817 |
| 1s_bwa_strelka_m≥3 | 530 | 1 | 629 | 0.998 | 0.457 | 0.627 |
| 2s_bowtie_mutect_m≥1 | 1097 | 234 | 62 | 0.824 | 0.947 | 0.881 |
| 2s_bowtie_mutect_m≥2 | 1049 | 4 | 110 | 0.996 | 0.905 | 0.948 |
| 2s_bowtie_mutect_m≥3 | 818 | 0 | 341 | 1.000 | 0.706 | 0.828 |
| 2s_bowtie_ss_m≥1 | 876 | 1151 | 283 | 0.432 | 0.756 | 0.550 |
| 2s_bowtie_ss_m≥2 | 598 | 189 | 561 | 0.760 | 0.516 | 0.615 |
| 2s_bowtie_ss_m≥3 | 412 | 17 | 747 | 0.960 | 0.355 | 0.519 |
| 2s_bowtie_strelka_m≥1 | 1023 | 175 | 136 | 0.854 | 0.883 | 0.868 |
| 2s_bowtie_strelka_m≥2 | 970 | 23 | 189 | 0.977 | 0.837 | 0.901 |
| 2s_bowtie_strelka_m≥3 | 752 | 5 | 407 | 0.993 | 0.649 | 0.785 |
| 2s_bwa_mutect_m≥1 | 1100 | 251 | 59 | 0.814 | 0.949 | 0.876 |
| 2s_bwa_mutect_m≥2 | 1021 | 10 | 138 | 0.990 | 0.881 | 0.932 |
| 2s_bwa_mutect_m≥3 | 807 | 0 | 352 | 1.000 | 0.696 | 0.821 |
| 2s_bwa_ss_m≥1 | 898 | 767 | 261 | 0.539 | 0.775 | 0.636 |
| 2s_bwa_ss_m≥2 | 784 | 155 | 375 | 0.835 | 0.676 | 0.747 |
| 2s_bwa_ss_m≥3 | 616 | 23 | 543 | 0.964 | 0.531 | 0.685 |
| 2s_bwa_strelka_m≥1 | 1007 | 696 | 152 | 0.591 | 0.869 | 0.704 |
| 2s_bwa_strelka_m≥2 | 970 | 15 | 189 | 0.985 | 0.837 | 0.905 |
| 2s_bwa_strelka_m≥3 | 554 | 1 | 605 | 0.998 | 0.478 | 0.646 |
| 3s_bowtie_mutect_m≥1 | 1091 | 227 | 68 | 0.828 | 0.941 | 0.881 |
| 3s_bowtie_mutect_m≥2 | 1046 | 3 | 113 | 0.997 | 0.903 | 0.947 |
| 3s_bowtie_mutect_m≥3 | 831 | 0 | 328 | 1.000 | 0.717 | 0.835 |
| 3s_bowtie_ss_m≥1 | 821 | 1149 | 338 | 0.417 | 0.708 | 0.525 |
| 3s_bowtie_ss_m≥2 | 579 | 204 | 580 | 0.739 | 0.500 | 0.596 |
| 3s_bowtie_ss_m≥3 | 475 | 15 | 684 | 0.969 | 0.410 | 0.576 |
| 3s_bowtie_strelka_m≥1 | 1023 | 177 | 136 | 0.853 | 0.883 | 0.867 |
| 3s_bowtie_strelka_m≥2 | 960 | 18 | 199 | 0.982 | 0.828 | 0.898 |
| 3s_bowtie_strelka_m≥3 | 755 | 3 | 404 | 0.996 | 0.651 | 0.788 |
| 3s_bwa_mutect_m≥1 | 1096 | 293 | 63 | 0.789 | 0.946 | 0.860 |
| 3s_bwa_mutect_m≥2 | 1037 | 5 | 122 | 0.995 | 0.895 | 0.942 |
| 3s_bwa_mutect_m≥3 | 812 | 0 | 347 | 1.000 | 0.701 | 0.824 |
| 3s_bwa_ss_m≥1 | 853 | 828 | 306 | 0.507 | 0.736 | 0.601 |
| 3s_bwa_ss_m≥2 | 761 | 147 | 398 | 0.838 | 0.657 | 0.736 |
| 3s_bwa_ss_m≥3 | 630 | 24 | 529 | 0.963 | 0.544 | 0.695 |
| 3s_bwa_strelka_m≥1 | 996 | 729 | 163 | 0.577 | 0.859 | 0.691 |
| 3s_bwa_strelka_m≥2 | 959 | 12 | 200 | 0.988 | 0.827 | 0.900 |
| 3s_bwa_strelka_m≥3 | 546 | 1 | 613 | 0.998 | 0.471 | 0.640 |

**Table S4** Performance scores of all centers approach for consensus cases. The notation "m" is used for multiple detection results. It indicates how many times a variant has been captured.

|  | **TP** | **FP** | **FN** | **Precision** | **Recall** | **F1** |
| --- | --- | --- | --- | --- | --- | --- |
| bowtie_mutect_m≥1 | 1109 | 708 | 50 | 0.610 | 0.957 | 0.745 |
| bowtie_mutect_m≥2 | 1098 | 23 | 61 | 0.979 | 0.947 | 0.963 |
| bowtie_mutect_m≥3 | 1093 | 3 | 66 | 0.997 | 0.943 | 0.969 |
| bowtie_mutect_m≥4 | 1079 | 2 | 80 | 0.998 | 0.931 | 0.963 |
| bowtie_mutect_m≥5 | 1058 | 1 | 101 | 0.999 | 0.913 | 0.954 |
| bowtie_mutect_m≥6 | 1013 | 1 | 146 | 0.999 | 0.874 | 0.932 |
| bowtie_mutect_m≥7 | 923 | 0 | 236 | 1.000 | 0.796 | 0.887 |
| bowtie_mutect_m≥8 | 837 | 0 | 322 | 1.000 | 0.722 | 0.839 |
| bowtie_mutect_m≥9 | 672 | 0 | 487 | 1.000 | 0.580 | 0.734 |
| bowtie_ss_m≥1 | 956 | 2412 | 203 | 0.284 | 0.825 | 0.422 |
| bowtie_ss_m≥2 | 834 | 724 | 325 | 0.535 | 0.720 | 0.614 |
| bowtie_ss_m≥3 | 769 | 365 | 390 | 0.678 | 0.664 | 0.671 |
| bowtie_ss_m≥4 | 698 | 205 | 461 | 0.773 | 0.602 | 0.677 |
| bowtie_ss_m≥5 | 632 | 108 | 527 | 0.854 | 0.545 | 0.666 |
| bowtie_ss_m≥6 | 579 | 56 | 580 | 0.912 | 0.500 | 0.645 |
| bowtie_ss_m≥7 | 534 | 17 | 625 | 0.969 | 0.461 | 0.625 |
| bowtie_ss_m≥8 | 468 | 9 | 691 | 0.981 | 0.404 | 0.572 |
| bowtie_ss_m≥9 | 362 | 1 | 797 | 0.997 | 0.312 | 0.476 |
| bowtie_strelka_m≥1 | 1075 | 714 | 84 | 0.601 | 0.928 | 0.729 |
| bowtie_strelka_m≥2 | 1032 | 53 | 127 | 0.951 | 0.890 | 0.920 |
| bowtie_strelka_m≥3 | 1013 | 30 | 146 | 0.971 | 0.874 | 0.920 |
| bowtie_strelka_m≥4 | 989 | 19 | 170 | 0.981 | 0.853 | 0.913 |
| bowtie_strelka_m≥5 | 965 | 15 | 194 | 0.985 | 0.833 | 0.902 |
| bowtie_strelka_m≥6 | 935 | 11 | 224 | 0.988 | 0.807 | 0.888 |
| bowtie_strelka_m≥7 | 849 | 4 | 310 | 0.995 | 0.733 | 0.844 |
| bowtie_strelka_m≥8 | 754 | 2 | 405 | 0.997 | 0.651 | 0.787 |
| bowtie_strelka_m≥9 | 635 | 2 | 524 | 0.997 | 0.548 | 0.707 |
| bwa_mutect_m≥1 | 1116 | 805 | 43 | 0.581 | 0.963 | 0.725 |
| bwa_mutect_m≥2 | 1101 | 25 | 58 | 0.978 | 0.950 | 0.964 |
| bwa_mutect_m≥3 | 1086 | 11 | 73 | 0.990 | 0.937 | 0.963 |
| bwa_mutect_m≥4 | 1066 | 4 | 93 | 0.996 | 0.920 | 0.956 |
| bwa_mutect_m≥5 | 1040 | 1 | 119 | 0.999 | 0.897 | 0.945 |
| bwa_mutect_m≥6 | 1005 | 0 | 154 | 1.000 | 0.867 | 0.929 |
| bwa_mutect_m≥7 | 898 | 0 | 261 | 1.000 | 0.775 | 0.873 |
| bwa_mutect_m≥8 | 821 | 0 | 338 | 1.000 | 0.708 | 0.829 |
| bwa_mutect_m≥9 | 679 | 0 | 480 | 1.000 | 0.586 | 0.739 |
| bwa_ss_m≥1 | 961 | 1788 | 198 | 0.350 | 0.829 | 0.492 |
| bwa_ss_m≥2 | 857 | 549 | 302 | 0.610 | 0.739 | 0.668 |
| bwa_ss_m≥3 | 820 | 280 | 339 | 0.745 | 0.708 | 0.726 |
| bwa_ss_m≥4 | 781 | 167 | 378 | 0.824 | 0.674 | 0.741 |
| bwa_ss_m≥5 | 763 | 112 | 396 | 0.872 | 0.658 | 0.750 |
| bwa_ss_m≥6 | 732 | 62 | 427 | 0.922 | 0.632 | 0.750 |
| bwa_ss_m≥7 | 661 | 27 | 498 | 0.961 | 0.570 | 0.716 |
| bwa_ss_m≥8 | 623 | 10 | 536 | 0.984 | 0.538 | 0.695 |
| bwa_ss_m≥9 | 533 | 3 | 626 | 0.994 | 0.460 | 0.629 |
| bwa_strelka_m≥1 | 1008 | 2255 | 151 | 0.309 | 0.870 | 0.456 |
| bwa_strelka_m≥2 | 1002 | 65 | 157 | 0.939 | 0.865 | 0.900 |
| bwa_strelka_m≥3 | 989 | 32 | 170 | 0.969 | 0.853 | 0.907 |
| bwa_strelka_m≥4 | 975 | 12 | 184 | 0.988 | 0.841 | 0.909 |
| bwa_strelka_m≥5 | 960 | 6 | 199 | 0.994 | 0.828 | 0.904 |
| bwa_strelka_m≥6 | 802 | 2 | 357 | 0.998 | 0.692 | 0.817 |
| bwa_strelka_m≥7 | 612 | 1 | 547 | 0.998 | 0.528 | 0.691 |
| bwa_strelka_m≥8 | 548 | 1 | 611 | 0.998 | 0.473 | 0.642 |
| bwa_strelka_m≥9 | 458 | 1 | 701 | 0.998 | 0.395 | 0.566 |

**Table S5** Performance scores of NeuSomatic Ensemble models labeled with within-center consensus results and ground truth (gt). The notation "m" is used for multiple detection results. It indicates how many times a variant has been captured.

|  | **TP** | **FP** | **FN** | **Precision** | **Recall** | **F1** |
| --- | --- | --- | --- | --- | --- | --- |
| FD_bwa_mutect_m≥1 | 546 | 118 | 191 | 0.822 | 0.741 | 0.779 |
| FD_bwa_mutect_m≥2 | 501 | 19 | 236 | 0.963 | 0.680 | 0.797 |
| FD_bwa_mutect_m≥3 | 451 | 5 | 286 | 0.989 | 0.612 | 0.756 |
| FD_bwa_ss_m≥1 | 546 | 118 | 191 | 0.822 | 0.741 | 0.779 |
| FD_bwa_ss_m≥2 | 507 | 20 | 230 | 0.962 | 0.688 | 0.802 |
| FD_bwa_ss_m≥3 | 462 | 8 | 275 | 0.983 | 0.627 | 0.766 |
| FD_bwa_strelka_m≥1 | 546 | 118 | 191 | 0.822 | 0.741 | 0.779 |
| FD_bwa_strelka_m≥2 | 485 | 10 | 252 | 0.980 | 0.658 | 0.787 |
| FD_bwa_strelka_m≥3 | 453 | 6 | 284 | 0.987 | 0.615 | 0.758 |
| FD_gt | 515 | 19 | 222 | 0.964 | 0.699 | 0.810 |
| IL_bwa_mutect_m≥1 | 667 | 47 | 70 | 0.934 | 0.905 | 0.919 |
| IL_bwa_mutect_m≥2 | 662 | 15 | 75 | 0.978 | 0.898 | 0.936 |
| IL_bwa_mutect_m≥3 | 650 | 11 | 87 | 0.983 | 0.882 | 0.930 |
| IL_bwa_ss_m≥1 | 666 | 47 | 71 | 0.934 | 0.904 | 0.919 |
| IL_bwa_ss_m≥2 | 664 | 21 | 73 | 0.969 | 0.901 | 0.934 |
| IL_bwa_ss_m≥3 | 654 | 11 | 83 | 0.983 | 0.887 | 0.933 |
| IL_bwa_strelka_m≥1 | 667 | 48 | 70 | 0.933 | 0.905 | 0.919 |
| IL_bwa_strelka_m≥2 | 663 | 26 | 74 | 0.962 | 0.900 | 0.930 |
| IL_bwa_strelka_m≥3 | 647 | 14 | 90 | 0.979 | 0.878 | 0.926 |
| IL_gt | 668 | 11 | 69 | 0.984 | 0.906 | 0.944 |
| NV_bwa_mutect_m≥1 | 693 | 31 | 44 | 0.957 | 0.940 | 0.949 |
| NV_bwa_mutect_m≥2 | 686 | 7 | 51 | 0.990 | 0.931 | 0.959 |
| NV_bwa_mutect_m≥3 | 673 | 1 | 64 | 0.999 | 0.913 | 0.954 |
| NV_bwa_ss_m≥1 | 693 | 32 | 44 | 0.956 | 0.940 | 0.948 |
| NV_bwa_ss_m≥2 | 682 | 7 | 55 | 0.990 | 0.925 | 0.957 |
| NV_bwa_ss_m≥3 | 670 | 0 | 67 | 1.000 | 0.909 | 0.952 |
| NV_bwa_strelka_m≥1 | 693 | 32 | 44 | 0.956 | 0.940 | 0.948 |
| NV_bwa_strelka_m≥2 | 687 | 10 | 50 | 0.986 | 0.932 | 0.958 |
| NV_bwa_strelka_m≥3 | 675 | 1 | 62 | 0.999 | 0.916 | 0.955 |
| NV_gt | 704 | 3 | 33 | 0.996 | 0.955 | 0.975 |

**Table S6** Performance scores of NeuSomatic Ensemble models labeled all centers approach consensus results and ground truth (gt). The notation "m" is used for multiple detection results. It indicates how many times a variant has been captured.

|  | **TP** | **FP** | **FN** | **Precision** | **Recall** | **F1** |
| --- | --- | --- | --- | --- | --- | --- |
| All_bwa_mutect_m≥1 | 546 | 118 | 191 | 0.822 | 0.741 | 0.779 |
| All _bwa_mutect_m≥2 | 500 | 15 | 237 | 0.971 | 0.678 | 0.799 |
| All _bwa_mutect_m≥3 | 510 | 18 | 227 | 0.966 | 0.692 | 0.806 |
| All _bwa_mutect_m≥4 | 508 | 18 | 229 | 0.966 | 0.689 | 0.804 |
| All _bwa_mutect_m≥5 | 501 | 12 | 236 | 0.977 | 0.680 | 0.802 |
| All _bwa_mutect_m≥6 | 507 | 16 | 230 | 0.969 | 0.688 | 0.805 |
| All _bwa_mutect_m≥7 | 492 | 11 | 245 | 0.978 | 0.668 | 0.794 |
| All _bwa_mutect_m≥8 | 486 | 7 | 251 | 0.986 | 0.659 | 0.790 |
| All _bwa_mutect_m≥9 | 435 | 2 | 302 | 0.995 | 0.590 | 0.741 |
| All _bwa_ss_m≥1 | 546 | 118 | 191 | 0.822 | 0.741 | 0.779 |
| All _bwa_ss_m≥2 | 505 | 15 | 232 | 0.971 | 0.685 | 0.804 |
| All _bwa_ss_m≥3 | 494 | 11 | 243 | 0.978 | 0.670 | 0.795 |
| All _bwa_ss_m≥4 | 510 | 19 | 227 | 0.964 | 0.692 | 0.806 |
| All _bwa_ss_m≥5 | 508 | 19 | 229 | 0.964 | 0.689 | 0.804 |
| All _bwa_ss_m≥6 | 491 | 12 | 246 | 0.976 | 0.666 | 0.792 |
| All _bwa_ss_m≥7 | 486 | 8 | 251 | 0.984 | 0.659 | 0.790 |
| All _bwa_ss_m≥8 | 485 | 7 | 252 | 0.986 | 0.658 | 0.789 |
| All _bwa_ss_m≥9 | 414 | 2 | 323 | 0.995 | 0.562 | 0.718 |
| All _bwa_strelka_m≥1 | 546 | 117 | 191 | 0.824 | 0.741 | 0.780 |
| All _bwa_strelka_m≥2 | 513 | 26 | 224 | 0.952 | 0.696 | 0.804 |
| All _bwa_strelka_m≥3 | 507 | 17 | 230 | 0.968 | 0.688 | 0.804 |
| All _bwa_strelka_m≥4 | 497 | 13 | 240 | 0.975 | 0.674 | 0.797 |
| All _bwa_strelka_m≥5 | 510 | 16 | 227 | 0.970 | 0.692 | 0.808 |
| All _bwa_strelka_m≥6 | 503 | 21 | 234 | 0.960 | 0.682 | 0.798 |
| All _bwa_strelka_m≥7 | 495 | 11 | 242 | 0.978 | 0.672 | 0.796 |
| All _bwa_strelka_m≥8 | 478 | 8 | 259 | 0.984 | 0.649 | 0.782 |
| All _bwa_strelka_m≥9 | 449 | 6 | 288 | 0.987 | 0.609 | 0.753 |
| FD_gt | 515 | 19 | 222 | 0.964 | 0.699 | 0.810 |
